# Evidence of *Peste des petits* Ruminants' Virus in Dromedary Camels in the Kingdom of Saudi Arabia between 2014 and 2016

**DOI:** 10.1155/2019/4756404

**Published:** 2019-11-12

**Authors:** Maged Gomaa Hemida, Hussain Mohammed Al-Ghadeer

**Affiliations:** ^1^Department of Microbiology, College of Veterinary Medicine, King Faisal University, Saudi Arabia; ^2^Department of Virology, Faculty of Veterinary Medicine, Kafrelsheikh University, Egypt; ^3^Department of Virology, Central Veterinary Diagnostic Laboratory, Ministry of Environment, Water and Agriculture, Riyadh, Saudi Arabia

## Abstract

Infection with the *Peste des petits *ruminants virus (PPRV) is a highly devastating viral infection of small ruminants. Dromedary camels live in close proximity of small ruminants in Arabian Peninsula (AP) and many other regions in the world. Little is known about the reasons behind continuous PPRV emergence in Saudi Arabia (KSA). Our objective was to test some dromedary camel population across the kingdom for the presence of specific PPRV antibodies. Our results show detection of specific PPRV antibodies (2.92%) in sera of tested dromedary camels from the eastern and south regions of the KSA. Our results suggest the exposure of dromedary camels to PPRV infection. Thus, dromedary camels may play some important roles in the sustainability of PPRV in the small ruminants across the AP. This is the first study examined the nationwide prevalence of the PPRV in dromedary camels in the KSA.

## 1. Introduction

*Peste des petits* ruminants (PPR) virus (PPRV) infection is a major viral disease affecting small ruminants, especially in Africa and Asia. Direct contact between PPRV infected and naïve animals facilitate transmission through both respiratory and fecal-oral routes [1, 2]. The PPRV infection is associated with a wide range of clinical signs including high fever, nasal and lachrymal discharges, diarrhea, and pneumonia. Mortality due to severe PPRV infection is very common in small ruminants [3, 4]. The PPRV belongs to the family *Paramyxoviridae*, sub-family Paramyxovirinae, and the genus Morbillivirus [5, 6]. The viral genome is a linear, nonsegmented, negative-sense single-stranded RNA that is approximately 15,948 nucleotides in length [7, 8]. Only one PPRV serotype has been identified so far, four lineages (I–IV) of this serotype have been reported [9]. This molecular classification is mainly based on the partial sequences of both the (N) and the (F) proteins [10]. Lineage I has been reported on the Ivory Coast and Senegal in Africa. Lineage II has been reported in Nigeria [11], lineage III has recently been reported in several countries in Africa and the Middle East including Uganda, Ethiopia, Oman, and United Arab Emirates [12]. Lineage IV has been reported in many parts of the world, especially in Asian countries such as China, Turkey, India, and Kurdistan [13]. In the KSA, a seroprevalence study of PPRV infection was conducted in small ruminants during the early 1980s [14]. Additionally, RRPV outbreaks have occurred in sheep and goats in the Al-Hasa province of the KSA in the early nineties, as well as in 2002 [15]. The 2002 PPR outbreak was shown to result in 100% mortality among the affected animals [16]. Another large-scale seroprevalence study has also been conducted on targeted animals from 11 different locations around the central region of the KSA. The study reported anti-PPRV antibody prevalence rates of 36.59% and 55.09% among the tested sheep and goat populations, respectively [17]. More recently, a seroprevalence study in the three main regions across the countries of Al-Hasa, Riyadh, and Assir. This study found that 40%, 85%, and 80% of the tested animals were seropositive for PPRV antibodies in each of the regions, respectively [18]. Recently, detection of PPRV in a group of wild ruminants such as (Arabian gazelle) antibodies was reported by other colleagues [19]. PPRV has been monitored in the KSA for more than three decades, however, despite active vaccination campaigns with a Nigerian isolate of the virus (PPRV-Nig-75), many PPRV outbreaks are still being reported [20]. A recent study reported an outbreak of PPRV in a herd of dromedary camels transferred from Kuwait to the south region in Iran [21]. This study reported the typical clinical and necropsy findings of PPRV in the tested camels. This was in addition to the seroconversion of the survived animals to the virus by ELISA [21].

The main goal of the current study was conduct a seroprevalence study of PPRV in dromedary camels living in close contact with PPRV-positive small ruminant flocks.

## 2. Materials and Methods

### 2.1. Dromedary Camel Sera

**Figure 1:**
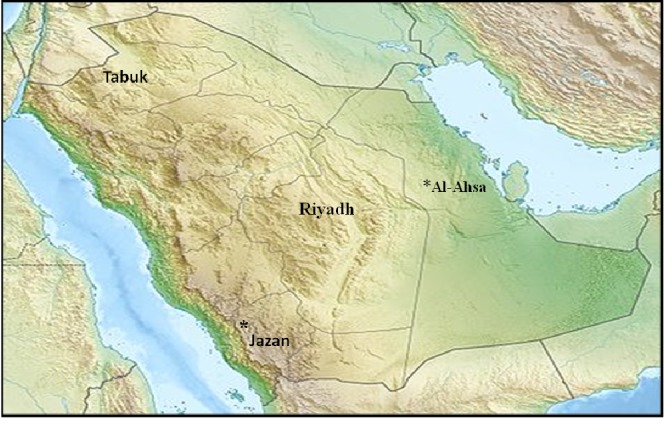
Geographical distribution of collected sera from dromedary camels across Kingdom of Saudi Arabia between 2014 and 2016. Map showing the geographical distribution of the suspected PPRV outbreaks in small ruminants across Saudi Arabia between 2014 and 2016.

A total of 370 camel serum samples were collected in the major regions of the KSA (Central, Riyadh Eastern, Al-Hasa. Southern, Jazan and North, and Tabuk), Figure 1. Samples were collected from camels raised in close vicinity of small ruminants. Whole blood samples were collected without anticoagulant and left overnight at 4°C. Clotted blood samples were then centrifuged at 5000 RPM for 10 mins. Separated sera were collected and exposed to heat inactivation at 56°C for 30 min. Sera were then stored at −20°C for further testing.

### 2.2. Enzyme-Linked Immunosorbent Assay (ELISA)

The collected dromedary camel sera samples (*n* = 370) were tested for the presence of anti-PPRV antibodies using commercial ID Screen® PPR Competition–IDVet kits (PPRC-10P). We used the ELISA technique as previously described earlier [13] and according to the kit instructions.

## 3. Results

Dromedary camel sera (*n* = 370) were collected from animals around four major cities in the KSA (Riyadh, Al-Hasa, Jazan, and Tabuk). These four cities represent the four major regions in the Kingdom (Central, Eastern, Southwest, and North) (Table 1). We tested sera of dromedary camels from those four regions in KSA (central, eastern, south and north). The tested animals lived in close contact with PPRV-positive small ruminant flocks, as confirmed by RT-PCR. Our results showed 11 out of 370 (2.997%) dromedary camel serum samples tested had high antibody titers (Table 1). Ten out of 153 (6.53) tested samples from Jazan were positive. Meanwhile, only one sample out of 93 tested (1.07) was positive from Al-Hasa region in the eastern region of KSA (Table 1).

### 3.1. Statistical Analysis

We conducted both a descriptive and inferential statistical analysis for our samples. We used Chi-square to test the significance of the difference between frequencies. Descriptive statistical analysis conducted to analyses the basic demographics. A *p*-value cut off point of 0.05 at 95% CI used to determine statistical significance. We used the Statistical Packages for Social Sciences [SPSS] version 21.

## 4. Discussion

Involvement of dromedary camels in the evolution and transmission of PPRV is inconsistent. Some studies have reported the absence of PPRV antibodies in sera of dromedary camels in the Canary Islands and the KSA [17]. However, recent studies from Nigeria and Iran reported a seroprevalence of anti-PPRV antibodies in dromedary camels [11, 21]. Supporting these later studies, other studies done in Sudan and Iran have reported that dromedary camels can be infected with PPRV, which leads to the typical clinical signs of PPRV and lesions in the affected animals [11, 21]. Other recent studies suggested that the PPRV host range is increased by including new species such as dromedary camels. This notion was supported by a recent study that found that an introduction of some Asian PPRV lineages in Africa, particularly in Morocco and Sudan [9, 11]. We conducted a nationwide serosurveillance targeting study to examine different dromedary camel herds across the KSA. Our data show that 11/370 serum samples were positive (2.97%) for anti-PPRV antibodies (Table 1).

Interestingly, 10 PPRV positive samples were from Jazan in the southern part of the country. This outcome may be related to the fact that this region shares a border Yemen, a PPR endemic country. An outbreak of the PPRV in dromedary camels in Iran confirms the potential roles of dromedary camels in the life cycle of the PPRV [21]. More recently, one study conducted in Kenya revealed that dromedary camels could be infected with the PPRV. Animals in this study showed typical PPRV clinical signs as shown by small ruminants [22]. There are no active vaccination campaigns against PPRV infection in dromedary camels in the KSA. This outcome may be a contributing factor for PPRV infection in this animal species. These data suggest that dromedary camels may play an important role in sustaining the PPRV in the environment. Further studies are urgently needed to understand the dynamics and roles of PPRV on dromedary camels in the KSA. We believe that this study will have a strong impact on the global PPRV eradication campaigns, including those in the KSA.

## 5. Conclusions

Detection of PPRV specific antibodies in sera of dromedary camels without previous vaccination history suggests exposure of these animals to the virus through natural routes of infection.

## Figures and Tables

**Table 1:** Results of the seroprevelance of PPRV in dromedary camels in Saudi Arabia 2014–2016.

Region	City	No. tested samples	(+Ve)	(−Ve)	% (+Ve)	Chi square	*p* value
Central	Ryd	76	0	76	0	25.72	0.001
Eastern	Hasa	93	1	92	1.07		
South West	Jaz	153	10	152	6.53		
North	TBK	48	0	48	0		

Total		370	11	359	2.97		

Ryd; Riyadh, Hasa; Al-Hasa, Jaz; Jazan, TBK; Tabuk.

## Data Availability

The data used to support the findings of this study are available from the corresponding author upon request.

## Additional Points

*Highlights.* Molecular characterization of the circulating PPRV strains in KSA during 2014–2016 Seroprevalence of PPRV among dromedary camels present in close contact with positive PPRV infected small ruminant herds.
